# Supplementary figures and images for: Non-invasive monitoring of drug action: A new live in vitro assay design for Chagas’ disease drug discovery
Source: PLoS Negl Trop Dis. 2020 Jul 27;14(7):e0008487. doi: 10.1371/journal.pntd.0008487 (PMC7419005; doi:10.1371/journal.pntd.0008487)

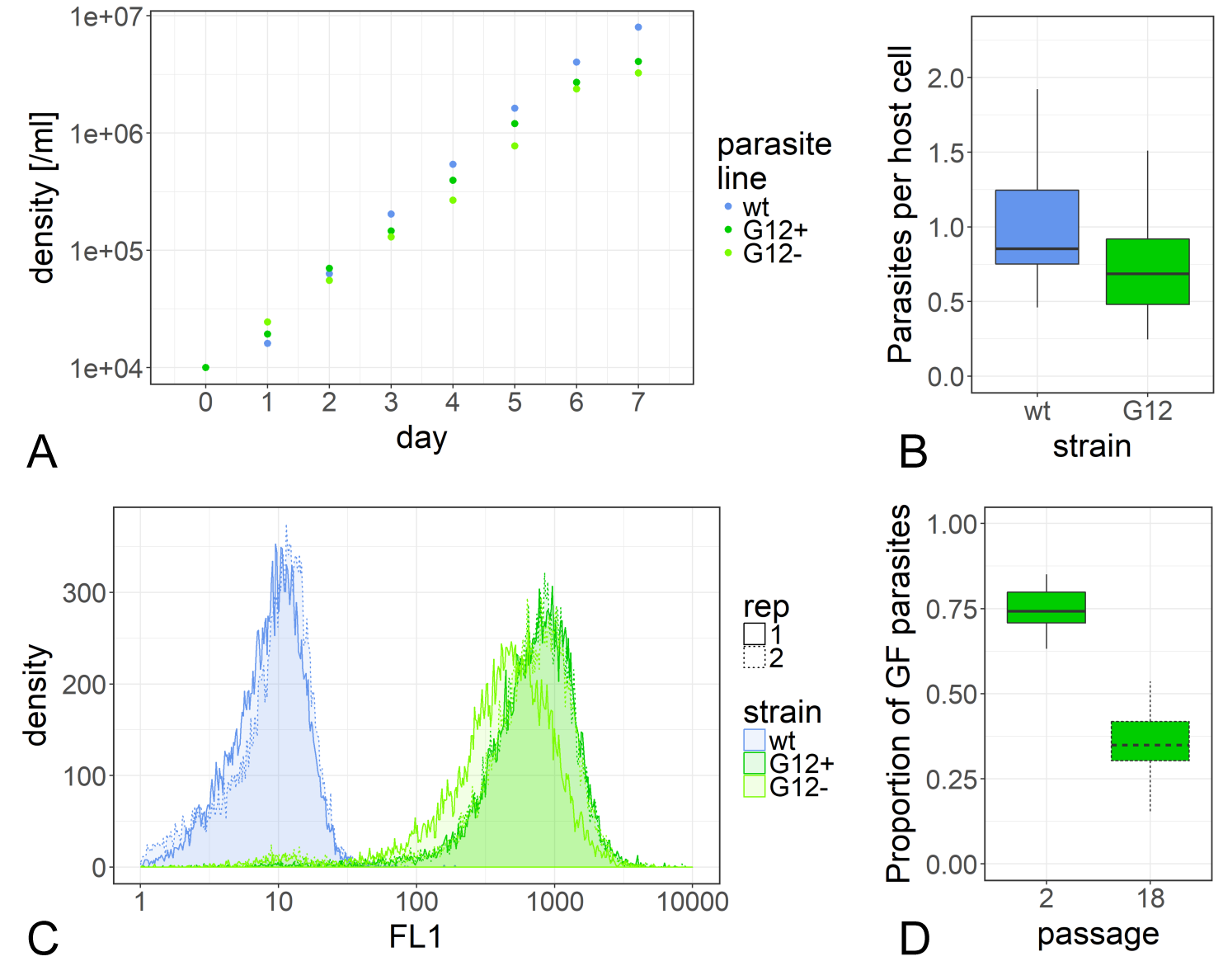

Supplement: S1 Fig — Epimastigote replication (A), trypomastigote infectivity (B), transgene expression stability in epimastigotes (C) and amastigotes (D) of the transgenic parasite line. Epimastigote density (A) was quantified daily after inoculum of 104 epimastigotes/ml using the Neubauer chamber. Wt denotes the STIB980 wildtype, G12+ the eGFP-expressing STIB980 line cultivated constantly in 500 μg/ml G418, and G12- the eGFP-expressing STIB980 line cultivated for 5 months without any antibiotic selection pressure. (B) Infectivity was measured using high-content microscopy of Hoechst-stained ePMM infected with the MOI 5:1 for 48 h. (C) Phenotypic transgene stability was measured in epimastigotes by flow cytometry in two replicates. The geometric mean of the fluorescence level of the parasite population and the proportion of green fluorescent parasites were determined (S1 Table). (D) In amastigotes, phenotypic transgene stability was measured by simultaneous comparison of ePMM infected for 5 days with a MOI 5:1 using trypomastigotes, which have either been passaged weekly 2 or 18 times in a Mef culture. (TIF) [file pntd.0008487.s001.tif]

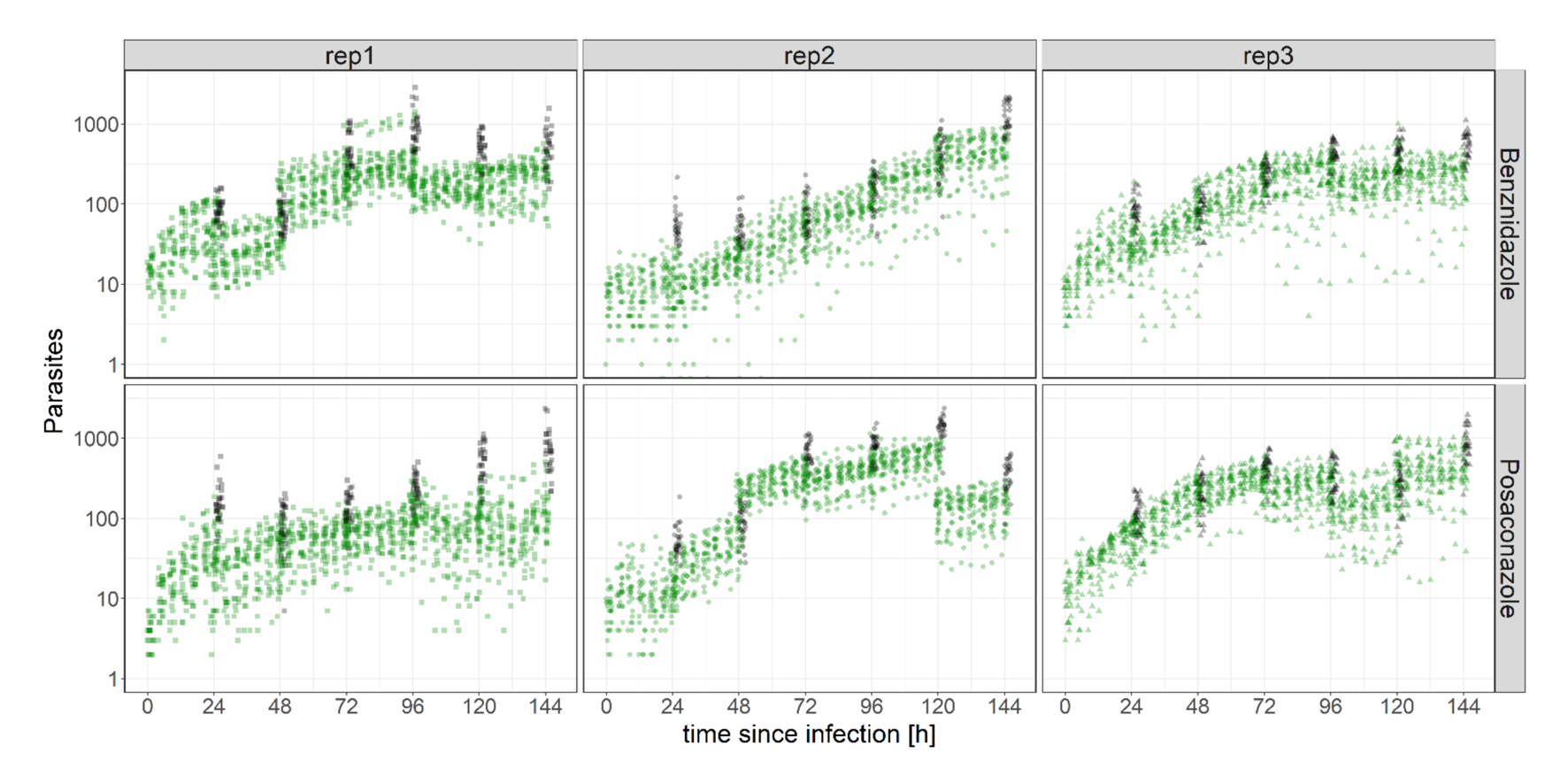

Supplement: S2 Fig — Parasite numbers per image from untreated wells of all biological replicates from live imaging (green, detected as GFP positive parasites) and fixed imaging (black, detected as kinetoplasts). (TIF) [file pntd.0008487.s002.tif]

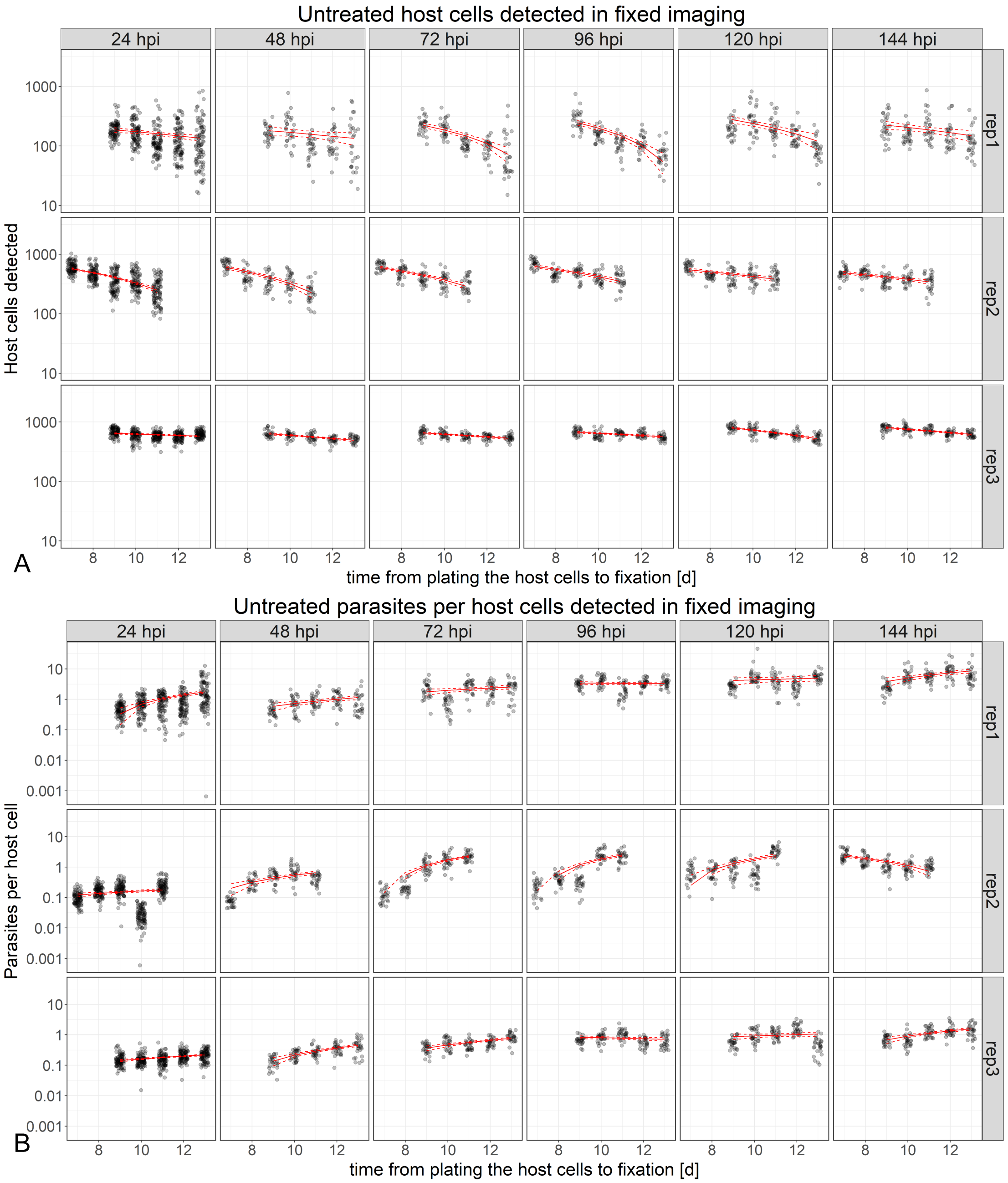

Supplement: S3 Fig — Host cell numbers per image (A) and parasites per host cell numbers per image (B) from untreated wells of all biological replicates from fixed imaging (black, parasites detected as kinetoplasts) in relation to time passed between plating the host cells and fixing the plate. Linear models of the correlation and their 95% interval are plotted in red. (TIF) [file pntd.0008487.s003.tif]

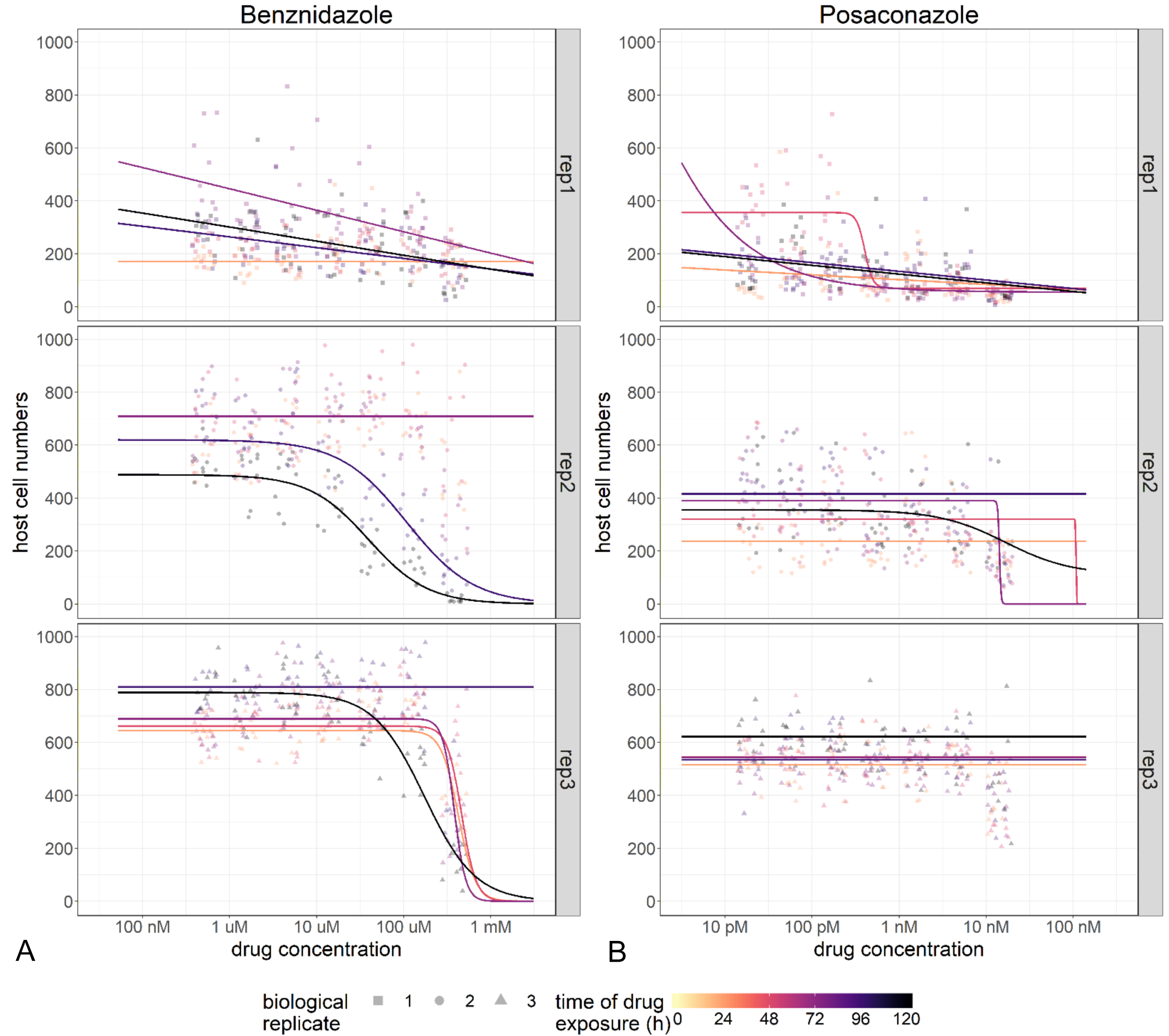

Supplement: S4 Fig — Host cell numbers per image at the given drug concentrations (of benznidazole (A), and posaconazole (B)) and the respective dose-response curves estimated using Equation 1 with the R package “drc” for all time points, at which a dose-response curve could be estimated. (TIF) [file pntd.0008487.s004.tif]

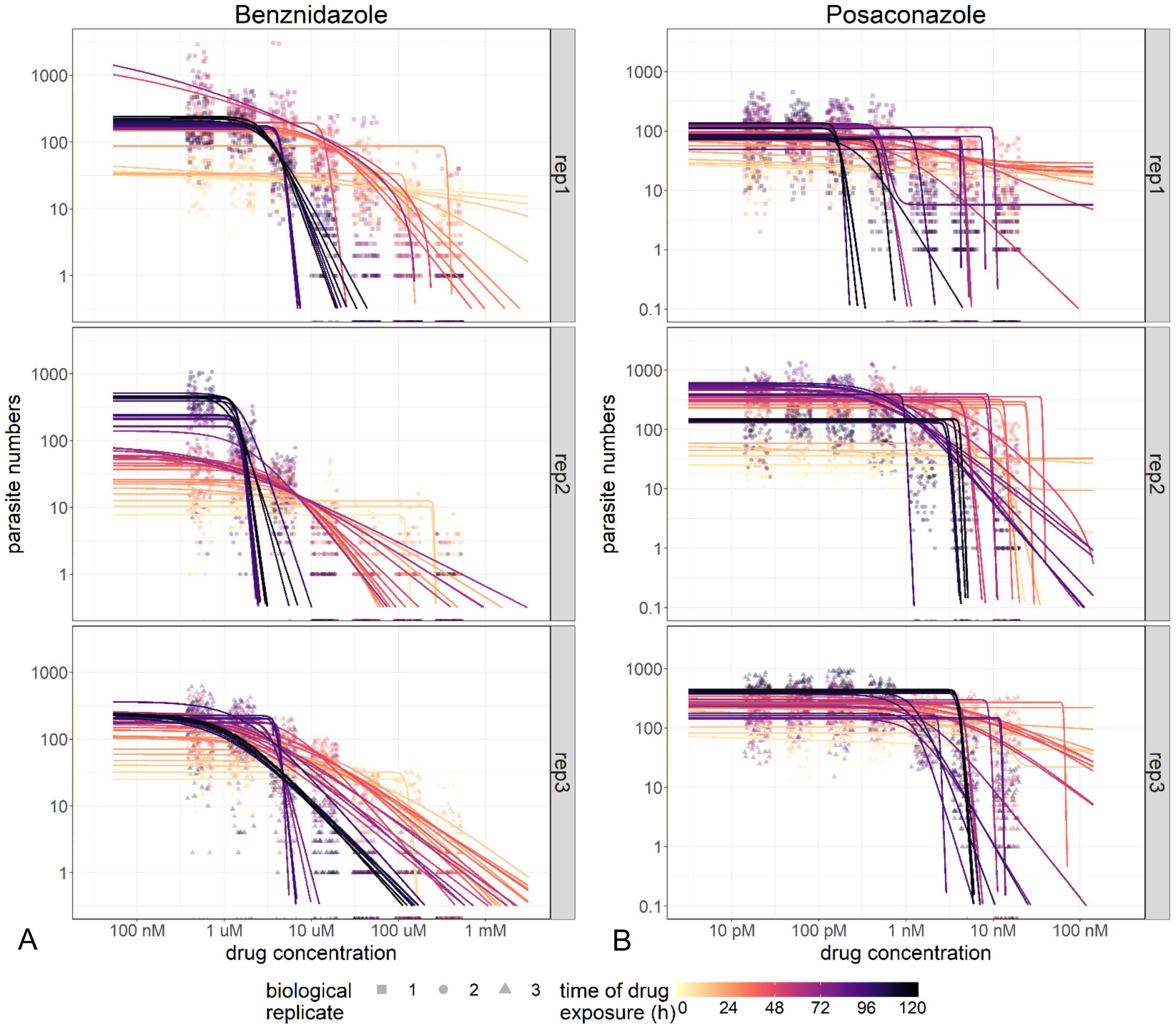

Supplement: S5 Fig — Parasite numbers per image (detected as green fluorescent parasites in the live imaging) at the given drug concentrations (of benznidazole (A), and posaconazole (B)) and the respective dose-response curves estimated using Equation 1 with the R package “drc” for all imaged time points. The y-axis in logarithmic scale to illustrate the parasite development over time. (TIF) [file pntd.0008487.s005.tif]
