# Supplementary material for: Non-invasive monitoring of drug action: A new live in vitro assay design for Chagas’ disease drug discovery
Source: PLoS Negl Trop Dis. 2020 Jul 27;14(7):e0008487. doi: 10.1371/journal.pntd.0008487 (PMC7419005; doi:10.1371/journal.pntd.0008487)
Supplement: S1 Table — Phenotypic transgene stability was measured in epimastigotes by flow cytometry in two replicates. The geometric mean of the fluorescence level of the parasite population and the proportion of green fluorescent parasites were determined. Wt denotes the T. cruzi STIB980 wildtype, G12+ the eGFP-expressing STIB980 line cultivated constantly in 500 μg/ml G418, and G12- the eGFP-expressing STIB980 line cultivated for 5 months without any antibiotic selection pressure. (DOCX) [file pntd.0008487.s006.docx]

Supp. Tab. 1. **Stability of transgene expression in *T. cruzi* epimastigotes.**

|  | **RFU (geometric mean)** | | **% Green fluorescent parasites** | |
| --- | --- | --- | --- | --- |
|  | **Replicate 1** | **Replicate 2** | **Replicate 1** | **Replicate 2** |
| **wt** | 8.3 | 8.6 | 0.110 | 0.367 |
| **G12+** | 640.0 | 670.0 | 98.9 | 98.3 |
| **G12-** | 580.0 | 790.0 | 96.4 | 96.1 |
